# The effects of suppressing inflammation by tofacitinib may simultaneously improve glycaemic parameters and inflammatory markers in rheumatoid arthritis patients with comorbid type 2 diabetes: a proof-of-concept, open, prospective, clinical study

**DOI:** 10.1186/s13075-023-03249-7

**Published:** 2024-01-04

**Authors:** Claudia Di Muzio, Ilenia Di Cola, Azadeh Shariat Panahi, Francesco Ursini, Annamaria Iagnocco, Roberto Giacomelli, Paola Cipriani, Piero Ruscitti

**Affiliations:** 1 Rheumatology Unit, Department of Biotechnological and Applied Clinical Sciences, University of L’Aquila, Delta 6 Building, PO box 67100, L’Aquila, Italy; 2 Medicine and Rheumatology Unit, IRCCS Istituto Ortopedico Rizzoli, Bologna, Italy; 3 Department of Biomedical and Neuromotor Sciences (DIBINEM), Alma Mater Studiorum University of Bologna, Bologna, Italy; 4 Academic Rheumatology Centre, Dipartimento di Scienze Cliniche e Biologiche Università di Torino - AO Mauriziano di Torino, Turin, Italy; 5 Clinical and Research Section of Rheumatology and Clinical Immunology, Fondazione Policlinico Campus Bio-Medico, Via Álvaro del Portillo 200, 00128 Rome, Italy; 6 Rheumatology and Clinical Immunology, Department of Medicine, University of Rome “Campus Biomedico”, School of Medicine, Rome, Italy

**Keywords:** Rheumatoid arthritis, Type 2 diabetes, Tofacitinib, Precision medicine, Therapy

## Abstract

**Background:**

A consistent connection has been increasingly reported between rheumatoid arthritis (RA), insulin resistance (IR), and type 2 diabetes (T2D). The β-cell apoptosis induced by pro-inflammatory cytokines, which could be exaggerated in the context of RA, is associated with increased expression pro-apoptotic proteins, which is dependent on JAnus Kinase/Signal Transducer and Activator of Transcription (JAK/STAT) activation. On these bases, we aimed to evaluate if the administration of tofacitinib, a potent and selective JAK inhibitor, could simultaneously improve glycaemic parameters and inflammatory markers in patients with RA and comorbid T2D.

**Methods:**

The primary endpoint was the change in the 1998-updated homeostatic model assessment of IR (HOMA2-IR) after 6 months of treatment with tofacitinib in RA patients with T2D. Consecutive RA patients with T2D diagnosis were included in this proof-of-concept, open, prospective, clinical study, which was planned before the recent emergence of safety signals about tofacitinib. Additional endpoints were also assessed regarding RA disease activity and metabolic parameters.

**Results:**

Forty consecutive RA patients with T2D were included (female sex 68.9%, mean age of 63.4 ± 9.9 years). During 6-month follow-up, a progressive reduction of HOMA2-IR was observed in RA patients with T2D treated with tofacitinib. Specifically, a significant effect of tofacitinib was shown on the overall reduction of HOMA2-IR (*β* =  − 1.1, *p* = 0.019, 95%CI − 1.5 to − 0.76). Also, HOMA2-β enhanced in these patients highlighting an improvement of insulin sensitivity. Furthermore, although a longer follow-up is required, a trend in glycated haemoglobin reduction was also recorded. The administration of tofacitinib induced an improvement in RA disease activity, and a significant reduction of DAS28-CRP and SDAI was observed; 76.8% of patients achieved a good clinical response. In this study, no major adverse events (AEs) were retrieved without the identification of new safety signals. Specifically, no life-threatening AEs and cardiovascular and/or thromboembolic events were recorded.

**Conclusions:**

The administration of tofacitinib in RA with T2D led to a simultaneous improvement of IR and inflammatory disease activity, inducing a “bidirectional” benefit in these patients. However, further specific designed and powered studies are warranted to entirely evaluate the metabolic effects of tofacitinib in RA patients with T2D.

## Introduction

The management of rheumatoid arthritis (RA), a chronic inflammatory disease, is strongly improved by present therapeutic strategies, but a high rate of comorbidities still burdens these patients leading to increased disability, morbidity, and mortality [1, 2]. In this context, multiple lines of evidence have increasingly reported a consistent connection between chronic inflammatory process and glucose derangement, as suggested by the elevated prevalence of type 2 diabetes (T2D) and insulin resistance (IR) in RA patients [3, 4]. IR is defined as the decreased sensitivity to metabolic actions of insulin, and it occurs early in the natural history of T2D and during RA; HOmeostasis Model Assessment of Insulin Resistance (HOMA-IR) is considered the most reliable and cost-effective surrogate measure in clinical settings. Interestingly, the pathogenic mechanisms behind IR and T2D may involve pro-inflammatory pathways in contributing to β-cell dysfunction and destruction [5]. In this context, previous studies described the simultaneous efficacy of immunomodulating therapies in RA patients with concomitant metabolic diseases [6, 7]. The inhibition of interleukin (IL)-1 and IL-6 was associated with simultaneous efficacy on RA and associated glucose derangement [8, 9]. In fact, the administration of these biological disease-modifying anti-rheumatic drugs (bDMARDs) led to the improvement of RA disease activity and glycaemic parameters, mostly IL-1 inhibition [10]. The latter induced a clinically relevant benefit in RA patients with T2D, who reached the therapeutic targets of both diseases [8]. These clinical observations suggested that the inflammatory mechanisms of T2D could be exaggerated in the context of RA, thus suggesting relevant therapeutic targets [10]. Remarkably, β-cell apoptosis induced by pro-inflammatory cytokines is associated with increased expression pro-apoptotic proteins, which is dependent on JAnus Kinase/Signal Transducer and Activator of Transcription (JAK/STAT) activation [11]. Mouse models have been instrumental in delineating the metabolic role of this signalling pathway in vivo suggesting the clinical usefulness of JAK/STAT inhibition on glucose derangement [11, 12]. Considering that JAK inhibitors (JAKis) are successfully used in RA, it could be theoretically hypothesised that these drugs could bidirectionally improve inflammatory signs and associated glucose abnormalities in patients with T2D. In fact, according to previous findings [8, 9], the administration of some RA-specific therapies could target the mechanisms of the concomitant cardiometabolic comorbidity providing a study model to be applied in this context. On these bases, we investigated whether the use of tofacitinib, a potent and selective JAKi, could simultaneously improve glycaemic parameters and inflammatory markers in RA patients with T2D in a proof-of-concept, open, prospective, clinical study.

## Methods

### Study design, setting, and patients

In 2019, we designed a 6-month proof-of-concept, open, prospective, clinical study to investigate the “bidirectional” role of tofacitinib in simultaneously improving glycaemic and inflammatory parameters in RA patients with T2D (*Comitato Etico Azienda Sanitaria Locale 1 Avezzano/Sulmona/L’Aquila, L’Aquila*, Italy; protocol number 0218811/19). This project was arranged before the recent emergence of safety data about tofacitinib [13–15]. In this study, RA patients with T2D were consecutively recruited among those undergoing treatment with tofacitinib in our centre and data were collected during scheduled visits. We aimed at investigating possible additional effects of tofacitinib on IR in RA patients with T2D, by using a “real life” design. Patients were considered eligible if all inclusion criteria were fulfilled: (i) adult age (≥ 18 years); (ii) RA classified according to 2010 ACR/EULAR [16]; (iii) diagnosis of T2D defined as a past diagnosis performed by a physician after the onset of RA or current treatment with antidiabetic medications (including oral antidiabetic drugs and insulin) started after the onset of RA; (iv) eligible for treatment with tofacitinib because moderate to severe disease activity despite treatment with conventional synthetic disease-modifying anti-rheumatic drugs (csDMARDs)/bDMARDs.

Dosage and route of administration of tofacitinib and each other medication were performed according to the manufacturer’s instructions. Any change of anti-rheumatic therapeutic strategy was not allowed, and concurrent csDMARDs and glucocorticoids (GCs), at low dosage (defined as daily dosage ≤ 7.5 [17]), were maintained throughout the follow-up period. Antidiabetic therapies were also retained unaltered during follow-up, unless an increase of glycated haemoglobin (HbA1c) > 1% or clinically relevant hypoglycaemia with the consequent exclusion from the study protocol.

The local Ethic Committee approved the study (*Comitato Etico Azienda Sanitaria Locale 1 Avezzano/Sulmona/L’Aquila, L’Aquila, Italy; protocol number 0218811/19*) which was conducted according to good clinical practice and the latest Declaration of Helsinki. Due to its nature, the study was conducted according to Italian Law concerning the “improvement of clinical practice” (*DM 17 Dicembre 2004*).

### Endpoints, clinical variables to be assessed, and bias

The primary endpoint was the change in HOMA2-IR after 6 months of treatment with tofacitinib. C-peptide was used instead of fasting insulin in order to avoid the interference of exogenous insulin in those patients treated with insulin therapy. Additional endpoints were also assessed: RA disease activity, physical disability, inflammatory markers, insulin sensitivity by HOmeostasis Model Assessment of β-cells (HOMA2-β), fasting values of glucose (FPG), HbA1c, body mass index (BMI), and lipid profile. HOMA2-IR and HOMA2-β were estimated by a dedicated tool available online (https://www.dtu.ox.ac.uk/homacalculator/).

The primary endpoint was investigated in 4 different subsets of patients to cover all possible clinical scenarios: inadequate responders to methotrexate (MTX), failures to a first bDMARD, failures to 2 or plus bDMARDs, intolerants to MTX who were treated with tofacitinib monotherapy.

Concerning possible biases, the main methodological problems were minimised by careful definition of each variable to be assessed. As above detailed, where possible, we maintained unaltered anti-diabetic therapy to minimise the possible biases due to change in drug dosages.

### Side effects

Safety profile was evaluated during all scheduled visit, and any suspected adverse event (AE) was recorded and coded according to the Medical Dictionary for Regulatory Activities system organ class classification. To minimise the risk of AEs, the following initiatives were undertaken: (i) a priori exclusion of all patients with absolute or relative contraindication of tofacitinib (i.e. known history of herpes zoster); ii. a priori exclusion of patients with potential contraindications to tofacitinib (i.e., impaired kidney and liver function).

### Sample size estimation

The primary outcome of our study was the change in HOMA2-IR after 6 months of treatment with tofacitinib. To this purpose, we have calculated a sample size of at least 7 patients to detect a difference between pre- and post-treatment values of at least 1 unit of change in HOMA2-IR, according to data published in studies investigating the effect of different bDMARDs on IR [18, 19]. The sample size has been adjusted to 10 patients to account for possible dropouts. Our study was specifically designed to evaluate a difference between pre- and post-treatment of HOMA-IR of 1 unit after 6 months of treatment, not on the proposed estimated means; therefore, we could not provide the standard deviation. For this calculation, the power was set at 80% and the confidence level at 95%. To cover all possible clinical scenarios, 4 groups of patients were assessed, collectively resulting in a planned sample size of 40 patients.

### Statistical analysis

Data generated from this study were analysed according to per-protocol and an intention-to-treat analysis in order to be as conservative as possible, maximising the collected results. The statistical analysis of primary endpoint was planned considering that the primary response variable was continuously distributed (HOMA2-IR) so that a linear mixed model was exploited accordingly. The analysis of secondary endpoints was performed by* t*-tests adjusting for the longitudinal design and multiple comparisons. Two-sided *P* values < 0.05 were considered statistically significant.

## Results

### Descriptive statistics of the final assessment

In this study, following the preliminary evaluation [20], 40 consecutive RA patients with T2D, who underwent treatment with tofacitinib, were included according to the estimated sample size (female sex 68.9%, mean age of 63.4 ± 9.9 years). Regarding RA features, 68.9% of patients showed rheumatoid factor and/or anti-citrullinated peptide antibodies, 55.8% of them had radiographic erosions, and 25.6% RA extra-articular manifestations. The disease duration was 7.1 ± 5.3 years, and 63.8% of patients had a disease duration ≥ 5 years. All included patients had moderate-to-high RA disease activity at the study inclusion. Among them, 60.5% of patients had high blood pressure, 41.9% showed a current or previous smoking habit, 27.9% were obese, and 18.6% had dyslipidaemia, 4.2% having clinical atherosclerosis. At the study beginning, HbA1c was 6.5 ± 3.9%. In our cohort, about 35% of patients were taking GCs, and 72.5% of them were simultaneously taking csDMARDs. In particular, tofacitinib was given with methotrexate in 67.4% of patients. Furthermore, 88.8% were currently treated with oral antidiabetic drugs, mainly metformin, and 11.2% with insulin. Additional clinical characteristics are reported in Table 1.

**Table 1:** Descriptive characteristics of included RA patients with T2D

*Clinical characteristics*	*40 RA patients with T2D*
*Demographics*	
Age, years, mean ± sd	63.4 ± 9.9
Female sex, %	68.9%
Smoking habit, %	41.9%
BMI, mean ± sd	28.2 ± 7.5
*RA features*	
RF and/or ACPA, %	69.8%
Radiographic erosions, %	55.8%
Extra-articular disease, %	25.6%
Disease duration, years, %	7.1 ± 5.3
Disease duration ≥ 5 years, %	63.8%
Disease duration ≤ 1 years, %	12.2%
DAS28-CRP, mean ± sd	4.1 ± 1.1
SDAI, mean ± sd	18.0 ± 8.3
ESR, mm/h mean ± sd	30.0 ± 15.0
CRP, mg/dl, mean ± sd	0.5 ± 0.4
Tender joints, mean ± sd	8.0 ± 5.0
Swollen joints, mean ± sd	4.1 ± 2.3
Patient Global Assessment, mean ± sd	5.9 ± 2.2
VAS pain, mean ± sd	6.2 ± 3.6
Evaluator Global Assessment, mean ± sd	5.7 ± 2.5
HAQ, mean ± sd	1.25 ± 1
*Comorbidity feature*	
T2D, %	100.0%
HbA1c %, mean ± sd	6.5 ± 3.9
HOMA2-IR, mean ± sd	1.8 ± 1.2
HOMA2-β, mean ± sd	72.3 ± 53.5
FPG, mg/dL, mean ± sd	136.4 ± 45.7
Total Cholesterol, mg/dL, mean ± sd	192.3 ± 68.6
Triglycerides, mg/dL, mean ± sd	146.6 ± 25.2
High blood pressure, %	60.5%
Obesity, %	27.9%
Dyslipidaemia, %	18.6%
Clinical atherosclerosis, %	4.2%
Previous Herpes Zoster, %	0.0%
Kidney disease, %	0.0%
Liver disease, %	0.0%
*Therapies*	
GCs, %	34.9%
GCs, mg/daily, mean ± sd	2.1 ± 1.6
csDMARD(s), %	72.5%
MTX, %	67.4%
Oral antidiabetic drugs, %	88.8%
Metformin, %	76.5%
Insulin, %	11.2%

*Abbreviations*: *BMI* Body Mass Index, *RF* Rheumatoid Factor, *ACPA* Anti Citrullinated Peptide Antibodies, *DAS28* Disease Activity Score using 28 joints, *CRP* C Reactive Protein, *SDAI* Simplified Disease Activity Index, *ESR* Erythrocyte Sedimentation Rate, *VAS* Visual Analogue Scale, *HAQ-DI* Health Assessment Questionnaire Disability Index, *T2D* Type 2 Diabetes, *HbA1c* glycated haemoglobin, *HOMA2-IR* HOmeostasis Model Assessment of Insulin Resistance, *HOMA2-β* HOmeostasis Model Assessment of β-cells, *FPG* fasting values of glucose, *GC* Glucocorticoids, *csDMARD* conventional synthetic Disease Modifying Anti-Rheumatic Drug, *MTX* Methotrexate

**Fig. 1:**
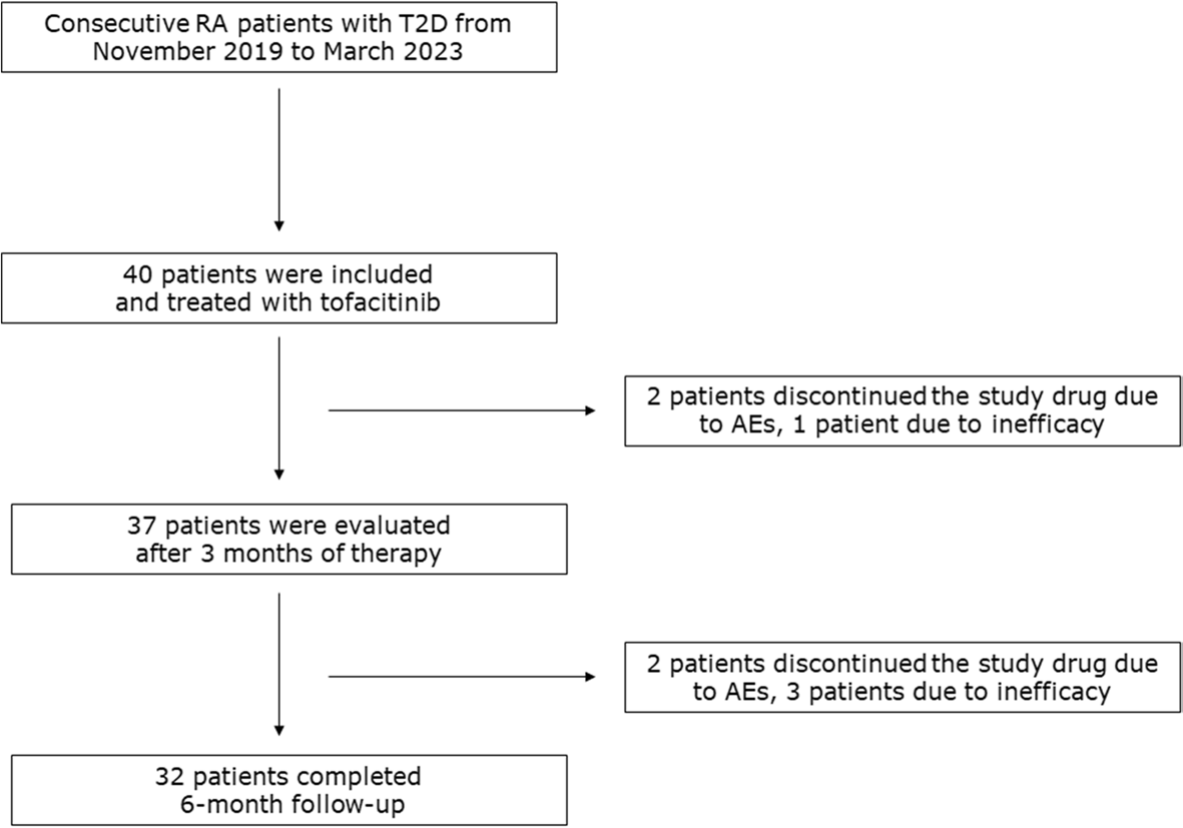
Study flow chart. Collectively, 40 patients were enrolled and 32 patients completed a 6-month follow-up; 4 discontinued the drug due to inefficacy and 4 due to adverse events

Collectively, 32 patients completed a 6-month follow-up (Fig. 1), 4 discontinued the drug due to inefficacy, and 4 due to AEs (better detailed below). An increase in HbA1c > 1% or clinically relevant hypoglycaemia was not recorded during the whole study follow-up.

### Tofacitinib improved IR and disease activity in patients with RA and T2D

**Fig. 2:**
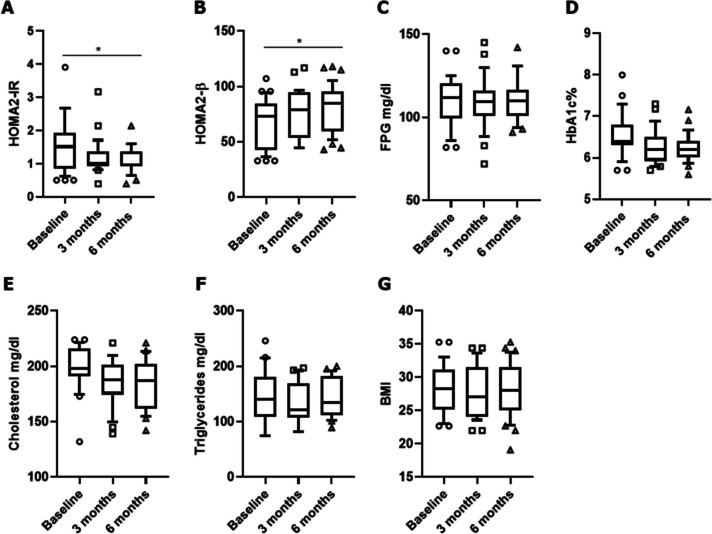
Metabolic endpoints following the administration of tofacitinib in RA patients with T2D. During the 6-month follow-up, a progressive reduction of insulin resistance was observed in RA patients with T2D treated with tofacitinib together with an improvement of insulin sensitivity. A trend in glycated haemoglobin reduction was also recorded. No significant effects were retrieved on fasting plasma glucose, cholesterol, triglycerides, and body mass index. At baseline, 40 patients were evaluated, 37 after 3 months, and 32 after 6 months, respectively. **A** HOMA2-IR, homeostasis model assessment of insulin resistance; **B** HOMA2-β, homeostasis model assessment of β-cell function; **C** FPG, fasting plasma glucose; **D** HbA1c, glycated haemoglobin; **E** cholesterol; **F** triglycerides; **G** BMI, body mass index

During the 6-month follow-up, a progressive reduction of HOMA2-IR was observed in RA patients with T2D treated with tofacitinib (*p* = 0.025), as reported in Fig. 2A. Specifically, a significant effect of tofacitinib was shown on the overall reduction of HOMA2-IR (*β* =  − 1.1, *p* = 0.019, 95%CI − 1.5 to − 0.76) in age- and male-sex-adjusted linear mixed model. Paralleling with HOMA2-IR, also HOMA2-β enhanced (Fig. 2B) highlighting an improvement of insulin sensitivity in these patients treated with tofacitinib (*p* = 0.035). Furthermore, although a longer follow-up is required on this metabolic outcome, a non-significant trend in HbA1c reduction was also recorded in our cohort (*p* = 0.077) (Fig. 2D). FPG and lipid profile did not significantly change during the follow-up (Fig. 2C, E, F, respectively). Moreover, BMI did not modify, thus excluding a possible anorexigenic effect of tofacitinib in these patients (Fig. 2G). All these results about metabolic endpoints are reported in Fig. 2.

**Fig. 3:**
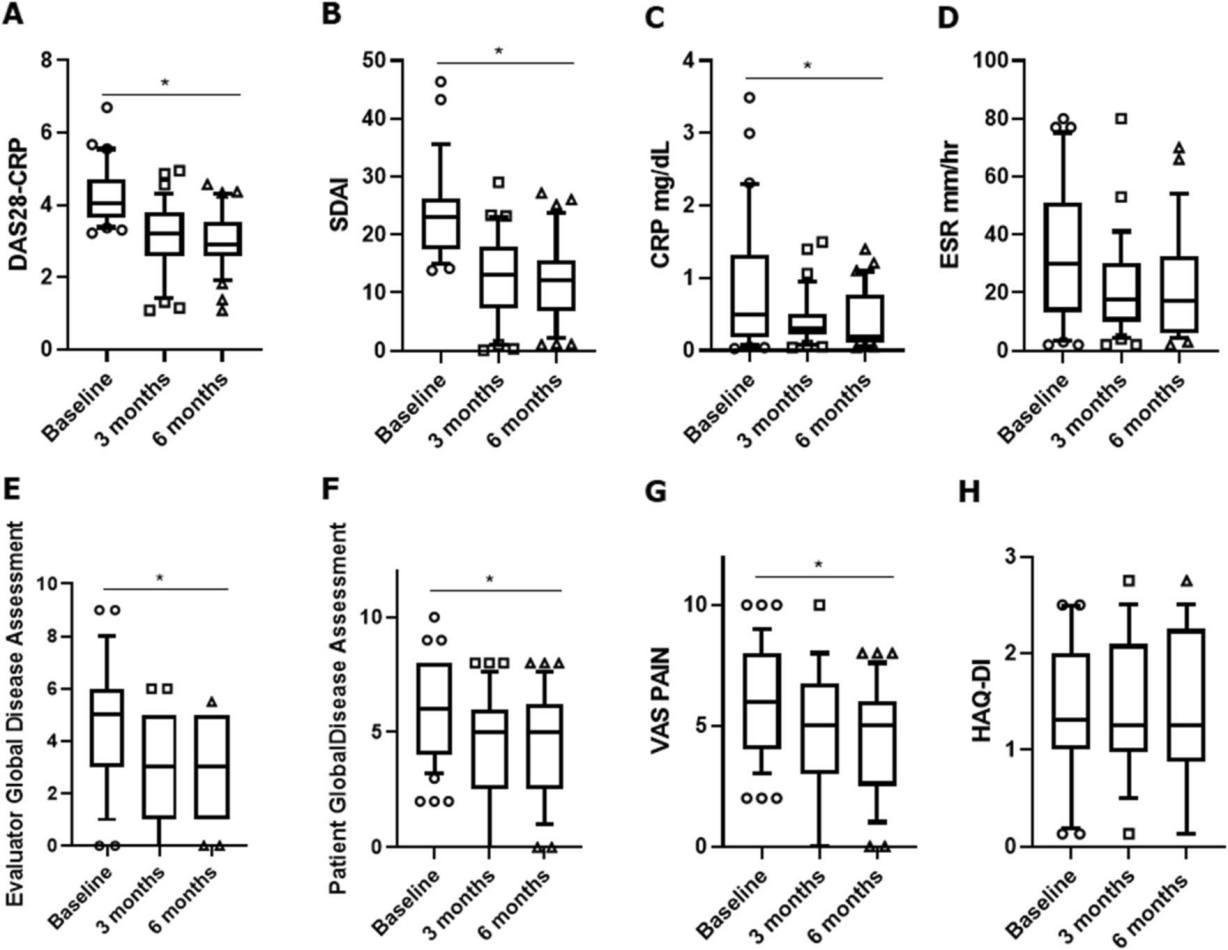
RA endpoints following the administration of tofacitinib in RA patients with T2D. The administration of tofacitinib induced an improvement of RA disease activity; a significant reduction was observed in values of DAS28-CRP, SDAI, C reactive protein, evaluator global disease assessment, patient global disease assessment, and VAS pain. At baseline, 40 patients were evaluated, 37 after 3 months, and 32 after 6 months, respectively. **A** DAS28, Disease Activity Score using 28 joints; **B** SDAI, simplified disease activity score; **C** CRP, C-reactive protein; **D** ESR, erythrocyte sedimentation rate; **E** evaluator global assessment; **F** patient global disease assessment; **G** VAS, Visual Analogue Scale; **H** HAQ-DI, Health Assessment Questionnaire Disability Index

In our cohort, the administration of tofacitinib induced an improvement of RA disease activity, and a significant reduction of DAS28-CRP (*p* < 0.001) and SDAI (*p* = 0.011) was observed, as reported in Fig. 3A and B, respectively. In addition, CRP (*p* = 0.035), patient global disease assessment (*p* = 0.018), and VAS pain (*p* = 0.024) significantly reduced (Fig. 3C, F, G, respectively). After 3 months of follow-up, 65.1% of patients achieved a low disease activity whereas 34.9% a remission. After a further 6 months of follow-up, 76.8% of patients achieved a low disease activity whereas 41.9% a clinical remission. We also assessed HAQ which did not change during the follow-up (Fig. 3H). All these findings about RA endpoints are summarised in Fig. 3.

Finally, considering baseline and 6-month values, we estimated possible correlations of ΔHOMA2-IR with both ΔDAS28-CRP and CRP. The 6-month improvement in HOMA2-IR significantly correlated with the parallel reduction of DAS28-CRP (*r* =  − 0.345, *p* = 0.031) and CRP (*r* =  − 0.298, p = 0.029). Also, we exploratively stratified these results about ΔHOMA2-IR according to male sex (male ΔHOMA2-IR =  − 0.9 ± 0.4 vs female ΔHOMA2-IR =  − 1.2 ± 0.6, *p* = 0.234) and the presence of obesity (obese ΔHOMA2-IR =  − 0.8 ± 0.5 vs non obese ΔHOMA2-IR =  − 1.3 ± 0.6, *p* = 0.158) but not significant results were obtained.

### Side effects

In this study, no major AEs were retrieved without the identification of new safety signals. Specifically, no life-threatening AEs were recorded. Specifically, 3 patients reported gastrointestinal intolerance manifesting with dyspepsia and diarrhoea and 2 patients described headache. All these side effects were completely reversed by tofacitinib discontinuation without long-term consequences. No major cardiovascular event (MACE) and/or thromboembolic episode were observed. Despite the improvement of glycaemic parameters, no episodes of hypoglycaemia were reported.

## Discussion

In this proof-of-concept, open, prospective, clinical study, the administration of tofacitinib in RA patients with T2D led to a simultaneous improvement of IR and inflammatory disease activity. Thus, JAK inhibition induced a “bidirectional” benefit in these patients with concomitant rheumatic and metabolic diseases.

Paralleling with the improvement of RA disease activity, in spite of the relatively small sample size, the relevance of inhibiting JAK/STAT pathway by tofacitinib could be suggested in RA patients with T2D. Our data mirrored available evidence on RA patients [21], but we assessed all patients with T2D, who were characterised by an established IR and a chronic glucose derangement. To contextualise these findings, in an experimental model of T2D, the administration of tofacitinib significantly decreased HOMA-IR and glycaemia, simultaneously improving glucose homeostasis, insulin secretion, and HOMA2-β [22]. In fact, a JAK/STAT pathway activation may inhibit the insulin signalling system by blocking the binding of insulin receptor substrates to the insulin receptor, thereby leading to IR, and contributing to T2D development [11, 12]. In addition, JAK/STAT signalling pathway mediates the action of a variety of hormones which have relevant effects on adipocyte development and function, influencing glucose homeostasis and development of IR [11, 12]. Furthermore, two inhibitors of JAK activity were identified in adipose tissue biology which may stably confer brown-like metabolic activity to white adipocytes [23]. Remarkably, by the induction of Uncoupling Protein 1, tofacitinib induced the phenomenon of adipocyte browning and/or the emergence of brown-like adipocytes in white adipose depots with consequent relevant metabolic benefits in the treatment of the metabolic diseases [23]. All these observations may suggest that the JAK/STAT signalling pathway may play a crucial role in the regulation of glucose derangement and obesity, thus suggesting its manipulation as a promising therapeutic strategy in this context. In fact, in RA patients with T2D, a possible vicious circle may be perpetuated by a rheumatoid inflammatory process, the presence of ACPA, deregulated glucose homeostasis, and adiposity [24–26]. RA metabolic alterations are shown to be linked with the degree of pro-inflammatory process, which may have as initial target inflammation of the adipose tissue in deregulating glucose as well as lipid homeostasis [24]. Interestingly, in RA patients, adipose tissue macrophages and crown-like structures are more represented and associated with the pro-inflammatory process and IR [25]. In addition, the presence of ACPA was also related to IR and altered adipocytokine profile [26], further reinforcing the idea of a strict connection between RA and glucose derangement. Thus, a better comprehensive control of inflammatory process and glucose alterations may improve the therapeutic management of RA patients with T2D, since the established IR of T2D may negatively influence the response of administered therapies [4, 6, 10].

Despite the short follow-up, major cardiovascular and thromboembolic events were not observed even if we included RA patients with T2D, who are considered at higher risk of accelerated atherosclerosis [27]. Recently, a clinical trial involving RA patients with cardiovascular risk factors showed a higher risk of MACE in patients randomised to receive tofacitinib than those receiving a tumour necrosis factor inhibitor (TNFi) [13]. These findings raised clinical concerns about the use of JAKi in these patients, also leading to changes in the recommendations for their administration. After that, post hoc analyses of these data have been carried out stratifying the patient population with different risks of MACE [14, 15]. Specifically, patients with a previous history of atherosclerotic disease, age ≥ 65 years, or smoking habit were at higher risk of MACE [14, 15]. In other patients, MACE risk did not appear to be different comparing patients receiving tofacitinib when compared with those with TNFi [14]. Our study was planned before the emergence of these data and we included patients at higher risk of MACE [3, 4]. However, the presence of T2D could represent a peculiar patient subgroup, possibly associated with specific disease mechanisms, suggesting the importance of an accurate stratification in guiding a benefit/risk assessment about the treatment with tofacitinib, according to patient clinical characteristics [20]. In addition, translational findings suggested that the administration of tofacitinib could improve some pathogenic mechanisms associated with accelerated atherosclerosis [28–30]. In fact, the inhibitory effects of tofacitinib have been recently shown on myofibroblast differentiation by transforming growth factor β and IL-6 [29]. Both in vitro and in vivo findings showed that tofacitinib may have anti-angiogenic properties [30]. Despite this being an unresolved issue, some studies suggested that aberrant angiogenesis may contribute to the growth of atherosclerotic plaque, leading to its destabilisation and consequent rupture [31, 32]. Anti-angiogenic agents could be effective in reducing atherosclerosis progression, although further confirmatory studies are needed [33, 34].

Our study is not without limitations and the derived results should be carefully interpretated. Although we provided some insights about the metabolic effects of tofacitinib, the single-centre design of our study may impair the generalisation of our data. Another limitation would be the lack of information about the titres of autoantibodies impairing possible further assessment of our results accordingly; as per study protocol, we registered only the presence/absence of these features. In addition, the relatively small number of assessed patients may advocate future studies specifically powered to assess these features. Therefore, the “hypothesis-generating” nature of our findings should be acknowledged in forming the basis for further confirmatory studies on harder metabolic endpoints. Alternatively, our data could also suggest the possible predictive role of T2D in predicting the clinical response to tofacitinib in RA patients to be specifically assessed. Taking together these findings, a more tailored management of RA could be advocated also including the evaluation of associated comorbidities [35, 36]. In addition, the development of a more accurate biomarker profile of these issues, also considering adipocytokine profile, may also improve the management of these patients with both RA and T2D [37, 38].

## Conclusions

In conclusion, the administration of tofacitinib in RA with T2D led to a simultaneous improvement of IR and inflammatory disease activity, inducing a “bidirectional” benefit in these patients with concomitant rheumatic and metabolic diseases. However, further specific designed and powered studies are warranted to entirely evaluate the metabolic effects of tofacitinib in RA patients with T2D.

## Data Availability

All data relevant to the study are included in the article.

## Abbreviations

RA: Rheumatoid arthritis
T2D: Type 2 diabetes
IR: Insulin resistance
HOMA-IR: HOmeostasis Model Assessment of Insulin Resistance
IL: Interleukin
bDMARDs: Biological disease-modifying anti-rheumatic drugs
JAK/STAT: JAnus Kinase/Signal Transducer and Activator of Transcription
JAKis: JAK inhibitors
csDMARDs: Conventional synthetic disease-modifying anti-rheumatic drugs
GCs: Glucocorticoids
MTX: Methotrexate
HOMA2-β: HOmeostasis Model Assessment of β-cells
HbA1c: Glycated haemoglobin
FPG: Fasting values of glucose
BMI: Body mass index
AE: Adverse event
MACE: Major cardiovascular event
TNFi: Tumour necrosis factor inhibitor
